# A Hypothesis: Linking Phase Separation to Meiotic Sex Chromosome Inactivation and Sex-Body Formation

**DOI:** 10.3389/fcell.2021.674203

**Published:** 2021-08-16

**Authors:** Yiding Xu, Huanyu Qiao

**Affiliations:** Department of Comparative Biosciences, University of Illinois at Urbana-Champaign, Urbana, IL, United States

**Keywords:** meiosis, phase separation, sex body, DNA damage response, heterochromatin, meiotic sex chromosome inactivation

## Abstract

During meiotic prophase I, X and Y chromosomes in mammalian spermatocytes only stably pair at a small homologous region called the pseudoautosomal region (PAR). However, the rest of the sex chromosomes remain largely unsynapsed. The extensive asynapsis triggers transcriptional silencing - meiotic sex chromosome inactivation (MSCI). Along with MSCI, a special nuclear territory, sex body or XY body, forms. In the early steps of MSCI, DNA damage response (DDR) factors, such as BRCA1, ATR, and γH2AX, function as sensors and effectors of the silencing signals. Downstream canonical repressive histone modifications, including methylation, acetylation, ubiquitylation, and SUMOylation, are responsible for the transcriptional repression of the sex chromosomes. Nevertheless, mechanisms of the sex-body formation remain unclear. Liquid-liquid phase separation (LLPS) may drive the formation of several chromatin subcompartments, such as pericentric heterochromatin, nucleoli, inactive X chromosomes. Although several proteins involved in phase separation are found in the sex bodies, when and whether these proteins exert functions in the sex-body formation and MSCI is still unknown. Here, we reviewed recent publications on the mechanisms of MSCI and LLPS, pointed out the potential link between LLPS and the formation of sex bodies, and discussed its implications for future research.

## Introduction

Meiosis is a special cell division that generates four gametes containing haploid genome. After DNA replication, germline cells enter meiotic prophase I, a prolonged G2-like stage. During prophase I, homologous chromosomes pair up, synapse, and exchange genetic fragments via a process known as homologous recombination. In mammalian spermatocytes, sex chromosomes (X and Y) are transcriptionally silenced by a mechanism known as meiotic sex chromosome inactivation (MSCI). Unlike autosomes that fully synapse between homologs at pachytene stage, the X and Y chromosomes only stably pair at the pseudo-autosomal region (PAR). Concurrent with MSCI, the silenced X and Y chromosomes are condensed and remodeled to form a distinct chromatin domain called sex body or XY body. Although the mechanism of MSCI has been extensively studied, how the sex body is formed and how the sex chromosomes are silenced are still unclear.

Increasing evidence suggests that DNA damage response (DDR) factors play important roles in MSCI (Namekawa, 2012; Turner, 2015). Proteins that typically respond to DNA double-strand breaks (DSBs) were found in the initiation steps of MSCI (Ichijima et al., 2012). Mutations in DDR factors, such as BRCA1 (Turner et al., 2004), phosphorylated histone variant H2AX (Fernandez-capetillo et al., 2003), ATM- and Rad3-related (ATR) kinase, ATR-activator TOPBP1 (Royo et al., 2013), and MDC1 (Ichijima et al., 2011), disrupt MSCI and sex-body formation, suggesting these factors play essential roles in silencing sex chromosomes. Recent publications on methyltransferase SETDB1 further linked the DDR with downstream histone modifications and transcriptional silencing (Hirota et al., 2018). However, many other linking proteins and histone modifications yet remain to be explored.

**FIGURE 1:**
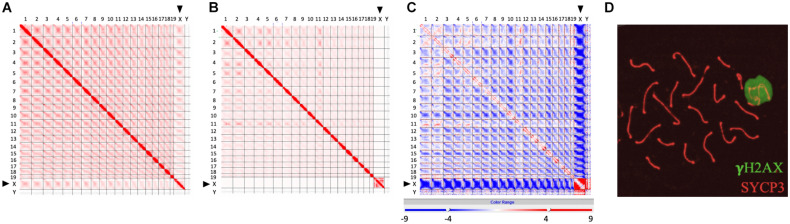
Isolated sex chromosomes at pachytene stage. **(A–C)** Hi-C contact maps showing reduced interactions between X chromosome and autosomes at pachytene stage, compared to zygotene stage. **(A)** Genome-wide Hi-C contact map generated from zygotene spermatocytes (modified from Patel et al., 2019). **(B)** Genome-wide Hi-C contact map generated from pachytene spermatocytes (modified from Patel et al., 2019). **(C)** Frequency difference map showing differences of contact frequency between **(A,B)** (pachytene stage vs. zygotene stage) (data from Patel et al., 2019). In Hi-C contact maps **(A,B)**, the intensity of each square represents the normalized number of contacts between a pair of chromosomes. Interactions between the X chromosome and other autosomes (1–19) at zygotene stage are more intensive than those at pachytene stage. In frequency difference map **(C)**, the intensity of each square represents the contact frequency difference between maps **(A,B)**. The intensive blue color shown between the X chromosome and autosomes indicates that contact frequency is significantly decreased in map **(B)** (pachytene stage), compared to map **(A)** (zygotene stage). **(D)** Images showing a sex body covered by γH2AX signal (green) in pachytene spermatocytes. Lateral elements of homologous chromosomes were stained by SYCP3 antibody (red).

Recently developed high-throughput chromosome conformation capture (Hi-C) has become a powerful tool to investigate the genome-wide chromatin conformation and interaction during meiosis (Ke et al., 2017; Alavattam et al., 2019; Patel et al., 2019; Wang et al., 2019). Based on the observation that the inactive X chromosome is isolated and distinctly regulated, Alavattam et al. (2019) first pointed out that the XY body and post-meiotic sex chromatin might form physically separated liquid droplets. We generated a frequency difference map to compare the Hi-C data of zygotene and pachytene chromosomes (sequencing data from Patel et al., 2019) and further confirmed that the sex body, as a separate territory, does not interact with other autosomes at pachytene stage (Figure 1; Page et al., 2012; Alavattam et al., 2019; Patel et al., 2019; Vara et al., 2019; Wang et al., 2019). Comparing to zygotene stage (Figure 1A), the interactions among autosomes are broadly reduced in pachytene stage, and contacts between the X chromosome and autosomes are completely lost (Figures 1B,C), indicating that sex chromosomes separate from autosomes at pachytene stage but not at zygotene stage. This is also described by Wang et al. (2019); they found that the X chromosome shows a unique chromatin configuration and largely loses topological association domains (TADs) and compartment features. These results are consistent with the observation of the distinct territory of sex chromosomes and the formation of the sex body (Figure 1D), which might largely abolish the interactions of sex chromosomes with autosomes.

While the factors involved in MSCI are well established, the mechanisms that physically separate sex chromosomes from autosomes have been an open question in the field. Wang et al. (2019) demonstrated there are more long-distance interactions within the X chromosome compared to autosomes, which may indicate X chromosomes are softer than stiff autosomes (Biggs et al., 2020). Unlike the rod-shaped autosomes, the territory of the X and Y chromosomes gradually becomes globe-shaped during sex-body formation. This unique sphere/egg-like shape of the sex body suggests surface tension at the liquid-liquid interface may minimize its surface area as what happens to oil droplets in water and phase-separated condensates in the nucleoplasm (Peng and Weber, 2019). Thus, we hypothesize that liquid-liquid phase separation (LLPS) may participate in the physical separation of sex chromosomes from the other autosomes. LLPS is a process in which a homogenous fluid demixes into two distinct liquid phases, driving the formation of various cellular compartments. Similar to how water is separated from oil in an oil-water mixture, many membraneless organelles, such as the nucleolus, Cajal body, and nuclear stress body, are possibly separated from the surrounding matter by LLPS (Aumiller et al., 2014; Razin and Gavrilov, 2020; Lafontaine et al., 2021). In addition, it has been implicated that LLPS drives the formation of heterochromatin regions that are transcriptionally inactive and enriched for repetitive sequences (Strom et al., 2017). However, how the phase separation influences gene expression is still a field that must be researched further. The X chromosome inactivation (XCI) silences one of the X chromosomes in female somatic cells and is also potentially related to LLPS (Cerase et al., 2019). The similarities between sex bodies and other membraneless organelles, heterochromatin, and XCI raise the possibility that LLPS also promotes the formation of sex bodies.

Here, we described the features of the sex body and reviewed the molecular mechanisms of MSCI. We also discussed current models for LLPS and its biological functions. Moreover, we hypothesized that LLPS could be the mechanisms for sex-body formation by comparing it with other phase-separating cellular condensates. Finally, we described several *in vivo* and *in vitro* experimental approaches to study LLPS proteins in sex bodies.

## Characteristics and Behaviors of the Sex Body

During meiotic prophase I in spermatocytes, the X and Y chromosomes undergo significant structural remodeling, compact into heterochromatin, and form the sex body. In the chromosomal spreads of early pachytene spermatocytes, the sex body is easily observed as a separated structure. During pachytene and diplotene stages, the sex body is deeply stained by Giemsa, possessing two joined and two separated ends of the X and Y chromosomes.

This large and darkly stained body was first observed in mammalian spermatocytes in the 1890s (Solari, 1974). Although it had been debated for a while whether a single X chromosome or both sex chromosomes are in this structure, later studies showed that both X and Y chromosomes are linked in this intranuclear body. Scientists first incorrectly named them “sex vesicles” because they assumed a surrounding membrane encloses sex chromosomes (Solari, 1969, 1974). However, after noticing that the “sex vesicle” appears not to be a membrane-bounded structure, researchers renamed it as the “sex body.” Solari (1974) also reviewed early studies on sex-body histochemistry. Initial assumptions claimed that this “sex vesicle” is enriched with RNA (Kaplan et al., 1956), however, Solari and Tres (1967) disproved this hypothesis by showing that RNA is only limited to the nucleolus that is associated with the sex body.

Unlike autosomes, X and Y chromosomes only partially synapse at their ends in a region called the pseudoautosomal region (PAR) where sex chromosomes share sequence homology (700 kb in mouse) (Perry et al., 2001). In mouse early pachytene cells, the synaptonemal complex (SC) loads on to 72% of the length of the Y axis and 22% of the X axis (Goetz et al., 1984). Furthermore, the chiasmata formed at the PAR is recognized at late pachytene stage (Burgoyne, 1982).

In summary, the sex body, a specialized and separated subnuclear structure, was identified and characterized in a set of early studies. Although the characteristics and behaviors of the sex body at different stages have been described, the molecular and biophysical processes underlying the sex-body formation within the nucleus are still unclear.

## Mechanisms of MSCI During Male Spermatogenesis

In recent years, the indispensable functions of MSCI during sex-body formation attract scientists’ attention. MSCI is a process that transcriptionally silences the X and Y chromosomes during meiotic prophase I of male spermatogenesis. After the zygotene-to-pachytene transition, any unsynapsed chromatin regions on autosomes are silenced by meiotic silencing of unsynapsed chromatin (MSUC) (Baarends et al., 2005; Turner et al., 2005). At pachytene stage, major portions of the X and Y chromosomes still keep unsynaped. Thus, the X and Y chromosomes compartmentalize to form a specialized nuclear domain and undergo transcriptional silencing (MSCI). Defects in MSCI cause misexpression of toxic sex-linked genes, such as *Zfy1/2* (Royo et al., 2010), that can eliminate the defective spermatocytes (Royo et al., 2015).

**FIGURE 2:**
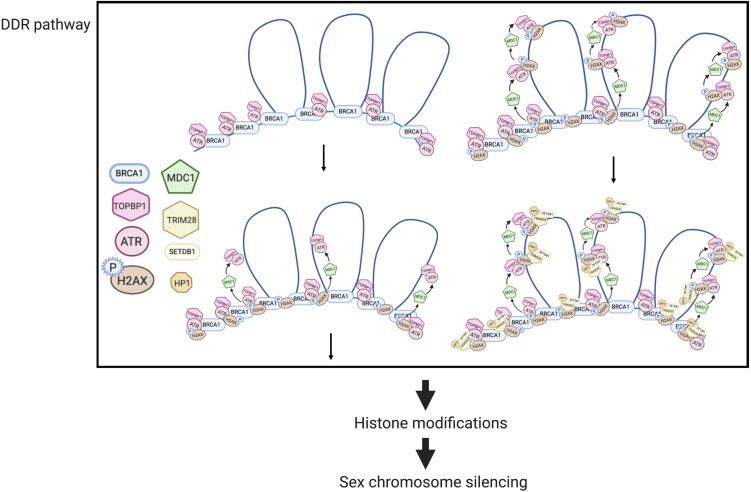
MSCI mechanisms in mouse spermatocytes (modified from Ichijima et al., 2011). In DDR responses, BRCA1 first loads onto the unsynapsed chromosome axis, then recruits the ATR-TOPBP1 complex, which further spreads onto the chromatin loops facilitated by MDC1. H2AX is then phosphorylated by ATR, forming γ-H2AX. Downstream of the DDR pathway, histone modifications mediate the transcriptional silencing of sex chromosomes.

**FIGURE 3:**
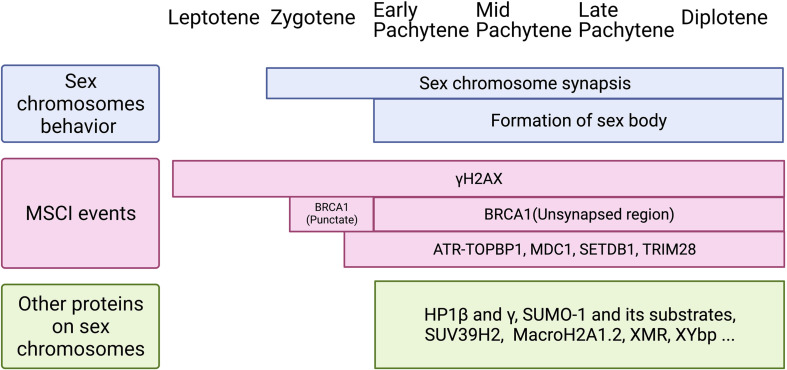
Schematic of sex chromosomes behavior, MSCI events, and loading of other proteins on the sex body during meiotic prophase I. **Top:** Sex chromosomes start to synapse at zygotene stage and keep synapsis at PAR until diplotene stage. The cytologically observed sex body forms at early pachytene and maintains until diplotene stage. **Middle:** Two waves of γH2AX are identified during meiotic prophase I. It loads onto the X chromosome as early as leptotene stage and to the Y chromosome at late zygotene stage. Then, it remains on the sex chromosomes axis and spreads genome-wide during pachytene stage, and maintains until diplotene stage. BRCA1 is first found on sex chromosomes at late zygotene stage, shown as punctate staining, and then remains on the unsynapsed axis from pachytene to diplotene. DDR factors, including ATR-TOPBP1, MDC1, SETDB1, and TRIM28, appear on sex chromosomes from late zygotene stage or early pachytene stage. **Bottom:** Other proteins found on the sex body during pachytene to diplotene stages. Although the function of these proteins is not clear yet, it is possible they are involved in histone modifications, phase separation, and the formation of the sex body.

Mechanisms of MSUC, and specifically, MSCI, have been extensively studied (Figure 2). Two sets of proteins, sensors and effectors, sequentially act to generate the silencing of sex chromosomes. In response to asynapsis, sensors initiate the signaling of asynapsis, such as HORMAD1 (Daniel et al., 2011), HORMAD2 (Wojtasz et al., 2012), and BRCA1 (Turner et al., 2004). Some effectors first localize to the unsynapsed axes, then spread to their associated chromatin loops, and mediate MSCI (Turner, 2015). ATR-TOPBP1 complexes are loaded onto the unsynapsed axes of the sex chromosomes and phosphorylated H2AX at Ser139 (Royo et al., 2013). Then, ATR-TOPBP1 complex further spreads to the chromatin loops directed by MDC1 (Ichijima et al., 2011). With the spreading of ATR-TOPBP1 complex, H2AX proteins along the chromatin loops are also phosphorylated by ATR, forming chromosome-wide γH2AX (Figure 2). γH2AX is essential for forming sex bodies and for inducing MSCI (Fernandez-capetillo et al., 2003). The sequential loading of MSCI-related proteins were summarized in Figures 2, 3 (Turner et al., 2004; Page et al., 2012; Turner, 2015).

MDC1 directly interacts with TOPBP1 and γH2AX during the amplification of phosphorylation signaling (Figure 2). In the absence of MDC1, ATR-TOPBP1, and γH2AX only restrict along the axes but not on the chromatin loops, indicating that the first-step axis localization of these DDR factors is MDC1-independent (Ichijima et al., 2011). Similarly, the failure of the chromosome-wide spreading of DDR factors also occurs in *H2ax-Y142A* mouse model. This mouse model has a point mutation on the phosphor-residue Y142 of H2AX that disrupts the interaction of γH2AX and MDC1 (Abe et al., 2020).

Although the loading of DDR proteins at early steps of MSCI has been well studied, what links the DDR network to the transcriptional silencing is largely unknown. Histone modifications, such as acetylation and methylation (Khalil et al., 2004), are involved in silencing sex chromosomes. A methyltransferase SETDB1 is recruited by γH2AX and mediates a gene-silencing-related histone modification – the trimethylation of histone H3 lysine 9 (H3K9me3) (Hirota et al., 2018). Tripartite motif-containing 28 (TRIM28) or KAP1 possibly bridges DDR to SETDB1 and regulates transcription (Hirota et al., 2018). However, more complicated epigenetic reprogramming might be involved in MSCI. For instance, the canonical histone H3.1 and H3.2 are replaced by H3.3 on unsynapsed sex chromosomes at mid-pachytene stage (van der Heijden et al., 2007). This replacement is accompanied by the loss of most histone post-translational modifications (PTMs), including temporally loss of H3K9me3 at pachytene stage (van der Heijden et al., 2007). This suggested there is a dramatic change in epigenetic modification and chromatin remodeling during both MSCI and MSUC.

## Sex-Body Formation and MSCI

The precise time course of the sex-body formation and the silencing of sex chromosomes have been long discussed (Figure 3). At early pachytene stage, after the initial sex-chromosome synapsis, MSCI-related proteins, such as SUMO1 (Small Ubiquitin Like Modifier 1), accumulate in the sex body (Rogers et al., 2004). At the same time, ubiquitin ligase UBR2 also localizes in the sex body and mediates histone H2A ubiquitination that is associated with transcriptional silencing of chromatin (Baarends et al., 2005). Page et al. (2012) demonstrated that silencing-related chromatin markers are present in the sex body before the transcriptional reactivation of autosomes. Thus, sex chromosomes may fail to reactivate at late zygotene stage rather than undergo inactivation at pachytene stage. Lau et al. (2020) also found transcriptional repression of the X chromosome starts before pachytene reactivation of autosomes by single-cell RNA-seq analysis. However, many other single-cell RNA-seq studies suggested that the initiation time of MSCI is pachytene stage (Chen et al., 2018; Green et al., 2018; Lukassen et al., 2018; Jung et al., 2019). The initiation-time difference may be caused by different clustering/staging approaches used in these RNA-seq studies.

As a separated, transcriptionally repressed chromatin region, the sex body harbors a range of specific proteins (Figure 3). DDR pathway proteins are found to be “sequestered” from the autosomes to the sex chromosome region at the initiation of MSCI (Abe et al., 2020). This “sequestration” effect could be explained by the high physicochemical affinity among these proteins (Handel, 2020). Besides those DDR factors, other proteins found in the sex body are also involved in heterochromatin formation. For instance, histone methyltransferase SUV39H2, a protein that modulates chromatin dynamics and usually distributes at the heterochromatin, accumulates to the sex-body region in pachytene spermatocytes (O’Carroll et al., 2000). Similarly, histone modification H3K9me3 plays an important role in meiotic heterochromatin assembly (Reuben and Lin, 2002; Hirota et al., 2018). H3K9me3 immunostaining signal increases in the sex-body region at early-mid pachytene transition, followed by temporal loss from mid/late-pachytene to late diplotene stage (van der Heijden et al., 2007; Page et al., 2012). Heterochromatin protein 1 (HP1), which binds to H3K9me3, has been shown to be related to chromatin condensation and transcriptional regulation. Two isoforms of the HP1 proteins, HP1β and HP1γ, decorate the entire sex body at late pachytene stage in human spermatocytes (Metzler-Guillemain et al., 2003), indicating their potential roles in condensing and silencing the sex chromosomes. However, more precise timing of HP1 loading should be explored, considering the complexity of histone modifications during MSCI. More interestingly, HP1-promoted phase separation has been proposed to facilitate the formation of chromatin sub-compartments (Erdel and Rippe, 2018) as well as transcriptional control (Hnisz et al., 2017).

Taken together, these studies provide evidence for potential close associations between MSCI and sex-body formation; however, the mechanisms underlying both processes need to be elucidated. Besides those well-studied proteins, the functions of many “sex-body” proteins are still uncertain. As reviewed by Handel (Handel, 2004), “sex-body” proteins, such as XMR (Escalier and Garchon, 2000), XYbp (Mazo and Pa, 2000), XY77, and ASY (Turner et al., 2000), all specifically localize in the sex body; whereas, the functions of these proteins remain unknown. To investigate the mechanisms of the sex-body formation, future studies should link heterochromatin formation and phase separation with transcriptional silencing and illustrate the timing of these events.

## Introduction to Liquid-Liquid Phase Separation (LLPS)

In the cell, LLPS is a biophysical phenomenon where macromolecules (such as proteins or nucleic acids) condense into a dense phase that demixes and creates multiple co-existing phases. LLPS has been proposed to drive the formation of many membraneless intracellular condensates and transform the way we think about subcellular organization. No membrane is observed surrounding the sex body, which leads to the idea that LLPS might separate sex chromosomes from autosomes to form the sex body. In a eukaryotic cell, intracellular space is divided into several membrane-bound organelles, each of which conducts different functions. Besides the canonical organelles bound by phospholipid bilayer membranes, many cellular compartments/condensates are not membrane-delimited, such as nucleoli, and still can separate themselves from other cellular components. Although these condensates are able to maintain their sizes and shapes, their assemblies are very dynamic and reversible. Environmental changes, such as composition, protein concentration, temperature, pH, and salt concentration, could all affect LLPS. Due to its dynamics, membraneless compartments can exert vital cellular functions and respond to environmental changes. For example, stress granules (SGs) are the LLPS-promoted assemblies of proteins and mRNAs under stress stimuli. They specifically function in the response to stress (Molliex et al., 2015).

**FIGURE 4:**
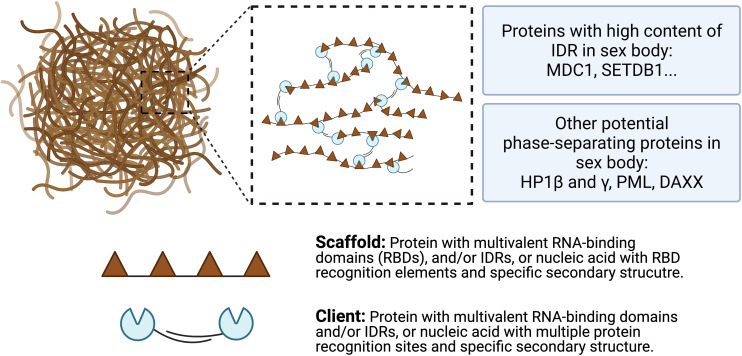
A model for multivalent interactions driving LLPS in sex body. Multivalent scaffold molecules (brown) recruit client molecules (blue) to form a phase-separated liquid droplet. Scaffolds are essential components of the phase-separated body while clients are dispensable and often present under certain conditions. Client molecules, which are not required for the condensate formation, bind to interaction domains/elements on scaffolds. Weak multivalent interactions between scaffolds and clients drive the LLPS. Potential phase-separating proteins are listed, including proteins with high content of IDR and other potential proteins.

So far, the detailed mechanisms of LLPS formation remain unclear. However, previous studies suggested that intermolecular multivalent interactions, including interactions between proteins, RNA, DNA, and membrane surface, are major drives of LLPS (Pak et al., 2016). A general model of scaffolds and clients is proposed to form the biomolecular condensates (Figure 4). Scaffolds are essential for the condensate formation. Protein scaffolds recruit various clients through intrinsically disordered regions (IDRs) while RNA scaffolds recruit clients via recognition elements of the RNA binding domains (RBDs) (Ditlev et al., 2018; Espinosa et al., 2020). Several characteristics of proteins and amino acids are responsible for forming phase-separated liquid droplets (Safaee and Michnick, 2016). For example, Wang et al. (2018) revealed that the LLPS of FUS-family proteins is driven by interactions between tyrosine and arginine residues. Liquid-droplet-forming proteins often contain low-complexity domains (LCDs). LCDs are composed of only a few different types of amino acids, either repeats of individual amino acids or short amino acid motifs. In addition, multiple-folded binding domains can interact with other peptides or nucleic acids; like LCD, proteins containing them have the ability to form phase-separated droplets (Banjade et al., 2015). Some theories have been proposed to mediate LLPS, such as the beta-amyloid formation model (Padrick and Miranker, 2002), the multivalent domain interaction network model (Falkenberg et al., 2013), and the polymer theory (Pappu et al., 2008), which have been reviewed by previous literature (Safaee and Michnick, 2016). However, how liquid droplets are assembled in a step-by-step manner still awaits for further investigations.

Phase separation is revealed to be responsible for the formation of heterochromatin (Strom et al., 2017). Similar to the heterochromatin regions, the sex body in male spermatocytes is also formed by heterochromatinization, suggesting a similar mechanism underlying the formation and silencing of both heterochromatin regions of autosomes and the sex body. To reveal these mechanisms, the common factors involved in phase separation within the two heterochromatic regions are still to be identified.

## LLPS in the Formation of Nuclear Compartmentalization

As a common phenomenon in the cell, LLPS is found to exert a variety of functions either at a single-cell level or during the development of organisms. A growing body of evidence suggests that LLPS forms phase-separated subcellular compartments (Table 1). In the nucleus, the formation of many membraneless organelles are facilitated by LLPS, such as nucleolus, Cajal body, clastosome, perinucleolar compartment, and polycomb body. Although the functions of some phase-separated condensates remain to be determined, the majority of them are involved in transcription, processing of rRNA, modification of snRNA, snoRNA, and other nuclear RNAs/RNPs.

**TABLE 1:** Membraneless compartments formed by LLPS in cells.

Compartment	Key components	Properties	Potential functions	References
P granules*	LAF-1, MEG-3, PGL-3	Spherical shape, undergoing fusion	Maintenance of germ cell totipotency, RNA processing, and storage	Brangwynne et al., 2009; Smith et al., 2016
Chromatoid body*	Ddx4	Cloud-like structure in male germ cells	piRNA-based gene silencing, RNA processing and storage	Kotaja and Sassone-Corsi, 2007
Balbiani body*	Xvelo	Solid-like structure in early-stage oocytes	Germline specification; protecting mitochondria and other organelles	Boke et al., 2016; Lei and Spradling, 2016
Nucleoli	FIB-1, DAO-5	Spherical shape, 1–4 per cell	Transcription and processing of rRNA, assembly of ribosome	Berry et al., 2015; Feric et al., 2016
Cajal body	Coilin, FMN	Spherical shape, 0–10 per cell	Modification and assembly of snRNA and snoRNA; trafficking of snRNP and snoRNP	Sawyer et al., 2019
Stress granules	hnRNPA1	100–200 nm in size	mRNA metabolism and translational control, involved in the pathogenesis of many diseases	Molliex et al., 2015
PML body	SUMO/SIM, TRF1/2	Spheres of 0.1–1.0 μm in diameter	DNA damage response, DNA repair, telomere homeostasis	Lallemand-Breitenbach and de Thé, 2010; Chung et al., 2011; Chang et al., 2018
Clastosome	19S and 20S proteasome	Doughnut-shaped	Protein degradation	Scherl et al., 2002
Polycomb body	Bmi1, Pc2, CBX2		Polycomb proteins mediated gene paring and silencing in *Drosophila*.	Vincenzo Pirrotta, 2012; Plys et al., 2019
P-bodies	EDC3, DDX6, LSM4, DCP2	8–10 nm in diameter	Primary piRNA processing	Luo et al., 2018
Centrosome	PLK1, CDK5RAP2/Cnn and Aurora-A	Consist of two barrel-shaped clusters of microtubules	Organization of microtubules and regulation of cytoskeletal structures	Mahen and Venkitaraman, 2012

*Indicates specific to germ cells.

Except RNA-containing condensates, LLPS participates in the formation of promyelocytic leukemia protein (PML) nuclear bodies (Boisvert et al., 2000), which are involved in multiple genome maintenance pathways (Chang et al., 2018; Corpet et al., 2020). The components of the PML nuclear bodies, SUMO1 and its substrates (PML and DAXX), are also found to accumulate in the sex body (Rogers et al., 2004).

## LLPS Drives Formation of Heterochromatin

Studies have shown that LLPS mediates the formation of distinct, multi-chromosomal, membraneless heterochromatin regions (Strom et al., 2017). Heterochromatin is characterized as a tightly packed form of DNA that enriched repetitive sequences. Trimethylated H3K9 works with HP1 to pack DNA. HP1 is identified as an intermediate protein that bridges chromatin to form heterochromatin. Proteins containing IDRs and low-complexity sequences often trigger LLPS; HP1α has both sequences and exhibits liquid demixing *in vitro* and *in vivo* (Strom et al., 2017). This phenomenon leads to the idea that the formation of heterochromatin is driven by LLPS (Larson et al., 2017). Properties and dynamics of phase-separated droplets are also present in the heterochromatin regions/domains, such as sensitivity to the change of hydrophobic interactions. This provides further evidence suggesting that LLPS may be involved in the formation of heterochromatin. Although we have known that transcriptional repression of heterochromatin regions is associated with phase separation, it is still unclear how genes in these regions are silenced, and whether phase separation is directly related to gene silencing.

## LLPS and X-Chromosome Inactivation

To balance the dosage of sex-linked genes in male and female cells, one of the X chromosomes in female is transcriptionally silenced by X chromosome inactivation (XCI) (Robert Finestra and Gribnau, 2017). Mechanisms underlying XCI have been studied for many decades in terms of protein recruitment, chromatin modification, and chromosome organization.

At the molecular level, X-inactive specific transcript (XIST), a long non-coding RNA (lnRNA), plays key regulatory roles in the repressive epigenetic modifications of XCI. A series of Xist-interacting proteins have been identified, which are involved in the transcriptional silencing and chromosome conformational changes. In the nucleus, XIST forms a cloud-like structure and works as a macromolecular platform to recruit its interactors (Minajigi et al., 2015). Histone modifiers, such as PRC1, PRC2 (Schoeftner et al., 2006; Mira-Bontenbal and Gribnau, 2016), and aurora kinase B (AURKB) (Hall et al., 2009), are identified as Xist-interactors and establish the repressive chromatin state. Cohesin proteins, including SMC1α,SMC3, RAD21, WAPL, and PDS5a/b, are also found in the Xist interactome. They are all involved in the structural reorganization of the inactive X chromosome (Xi) (Minajigi et al., 2015). CCCTC-binding factor (CTCF), a master regulator of genome architecture, also directly interacts with Xist RNA and mediates long-rang chromosomal interactions (Kung et al., 2015). Due to the actions of these proteins, the Xi is characterized by a variety of chromatin modifications (Pinter, 2016), including histone deacetylation (Belyaev et al., 1996), demethylation at histone H3 lysines 4 and 36 (Boggs et al., 2002), tri-methylation of lysine 27 in histone H3 (H3K27me3) mediated by PRC2 (Zhao et al., 2008), and monoubiquitination of lysine 119 in histone H2A (H2AK119ub1) mediated by PRC1 (de Napoles et al., 2004).

Revealed by genome capture technique, Hi-C, the active and inactive X chromosome show different topological conformations (Robert Finestra and Gribnau, 2017). The Xi chromosome is depleted of active/inactive compartments as well as topologically associating domains (TADs) (Filippova et al., 2005; Giorgetti et al., 2016; Pal et al., 2019). These chromosomal structural studies link the chromatin modifications with the chromosome organization as well as Xi transcriptional silencing, which makes the mechanism of XCI a classical example to study the epigenetic processes and gene regulation. Similarly, during meiosis in mammalian males, the X chromosome was found to be reorganized, isolated from all autosomes, and completely lose the compartment structure in pachytene stage (Patel et al., 2019). However, evidence showed that although both MSCI and XCI involve dramatic chromosome reorganization, the structures are distinct. For example, Xi displays two megadomains separated at the DXZ4 boundary (Giorgetti et al., 2016), which is not observed in sex chromosomes during male meiosis (Alavattam et al., 2019; Wang et al., 2019).

When comparing XCI in females and the silencing of sex chromosomes (MSCI) in males, some similarities should be aware of. First, high-throughput chromosome conformation capture (Hi-C) studies revealed TADs and compartments are lost during XCI and MSCI; the chromosome-wise transcriptional silencing of the X chromosome is a consequence of both processes (Giorgetti et al., 2016; Patel et al., 2019). Second, chromatin modifications, such as acetylation/deacetylation, methylation/demethylation, and ubiquitination, are utilized in both inactive chromatin regions. Third, more importantly, although there is still no direct evidence suggesting the involvement of phase separation in both processes, Cerase et al. (2019) summarized pieces of evidence showing that Xist assemblies resemble phase-separated condensates in size, morphology, and composition. Additionally, some binding partners of Xist have been shown to undergo LLPS and have a strong tendency for phase separation (Cerase et al., 2019). Considering the similarities between sex body and Xi, the involvement of LLPS in the formation of the sex body should also be considered.

## Associations Among Sex Chromosomes and Nucleolus

The inactive X chromosome was described as the “nucleolar satellites” by Barr and Bertram (1949), indicating the close association is present between Xi and nucleoli. Zhang et al. (2007) also found that 80–90% of Xi localize to the nucleolus during mid-to-late S phase. This association is confirmed by the genome-wide mapping of nucleolus-associated chromosomal domains (NADs) (Dillinger et al., 2017). Analysis of chromatin states of NADs demonstrated that NADs are mainly heterochromatic and lack active chromatin. The localization of Xi to the condensed perinucleolar compartment is proposed to play essential roles in establishing its epigenetic status and repressing its genes (Zhang et al., 2007; Pandya-Jones et al., 2020). Similarly, the association of sex chromosomes with nucleolus was also observed decades ago (Gates, 1939). Nucleolar masses detach from the nucleolar organization sites of their autosomal origins and migrate toward the sex body at mid pachytene stage (Tres, 2005). At late pachytene stage, a half-moon-shaped nucleolus wraps up and covers half the surface of the sex body. The thread-like granule layer of the nucleolus penetrates deeply into the chromatin part of the sex body (Solari, 1969). *In situ* hybridization has shown that sex chromosomes were found non-randomly distributed in the nucleus and close to the nucleolus (Weipoltshammer et al., 1996; Schöfer and Weipoltshammer, 2018). It is still unknown how the association between sex body/Xi and nucleolus forms (Handel, 2020). However, we have already known phase-separation drives the formation of the nucleolus (Lafontaine et al., 2021). If sex body and Xi share similar phase-separating properties with the nucleolus, the fusion of the phase-separated sex body/Xi and nucleolus might explain these two associations.

## Approaches and Tools to Study LLPS

Considering the similarity between sex body and other phase-separated condensates, we speculate that LLPS is the mechanism that drives the formation of the sex bodies. The results from Abe et al. (2020) suggested that the LLPS-mediated sex body functions as a sink to trap other proteins, which links this special structure with its potential functions. However, there is little research focusing on the roles of LLPS in sex-body formation. Here, we summarize several experimental approaches and tools that can be applied to study how LLPS drives sex-body formation.

It is known that only a small subset of proteins are able to undergo LLPS under specific conditions. Properties and sequences associated with these phase-separated proteins have been intensively studied. Two major types of proteins have been identified to form a network of interactions and promote LLPS. First, IDR-containing proteins are essential for phase separation. Certain polar and charged amino acids are often enriched in IDRs, including glycine (G), serine (S), glutamine (Q), proline (P), glutamic acid (E), lysine (K), and arginine (R). These charged amino acids enable various protein-protein and RNA-protein interactions to promote LLPS. In particular, IDR-containing proteins with low-complexity domains exhibit phase-separating properties. Second, the other type of phase-separated proteins is characterized by multiple folded domains that provide multivalent interactions with other proteins. For example, the SH3 domain covalently crosslinks with PRM ligands, driving phase transition (Li et al., 2012).

In common, both types of proteins mediate LLPS by multivalent interactions. Based on this property, whether a protein is able to undergo LLPS could be predicted by its primary sequence. Numerous analysis tools can be used to look for LLPS-promoting proteins, such as UniProt, BLAST, ProParam, and CIDER. In addition, LLPS predictors are generated based on searching disordered domains or regions. For example, MobiDB (Piovesan et al., 2021), D2P2, and DisMeta are powerful tools for disorder prediction. Moreover, several phase-separation predicting software have been developed. Pi-Pi predictor, Prion-like amino acid composition (PLAAC), and ZipperDB are designed for predicting the pi-pi contacts, prion-like domains, and fibril-forming segments, respectively. Alberti et al. (2019) compared and summarized the principles of different searching methods. Utilizing these tools and databases, meiotic proteins, especially those localizing to the sex body, could be screened and analyzed to determine whether they harbor phase-separating sequences and exhibit phase-separating properties. For example, using MobiDB, we obtained the disorder content of a list of meiosis-related proteins (Table 2), which helps identify phase-separation proteins in meiosis and providing the basis for future study of detailed characteristics. Among meiotic proteins we analyzed, although most of them are present in both sex chromosomes and autosomes, a DDR protein, 53BP1, accumulates in the XY body (Ahmed et al., 2007; Gupta et al., 2013; Lu et al., 2013). 53BP1 has a high content of IDR domains and has been shown to undergo phase separation (Kilic et al., 2019). In addition, MSCI-related protein, MDC1 (Ichijima et al., 2011), also shows a high percentage of disordered domains and is possibly involved in phase separation. Further *in vitro* and *in vivo* experiments should be conducted to investigate the potential roles of these two proteins in sex-body phase separation.

**TABLE 2:** IDR content information of meiosis-related proteins based on MobiDB.

Protein name	IDR content	Functions
Testis-specific H1 histone (H1t)	0.714	Associated with repressed chromatin domains in pachytene spermatocytes (Mahadevan et al., 2020)
Mediator of DNA damage checkpoint protein 1 (MDC1)	0.697	Functions in chromosome-wide silencing of the sex body (Ichijima et al., 2011)
TP53-binding protein 1 (53BP1)	0.624	DDR protein and localizes to the sex body (Lu et al., 2013)
Histone-lysine N-methyltransferase SETDB1	0.338	Links meiotic DDR to sex chromosome silencing (Hirota et al., 2018)
Pachytene checkpoint protein 2 homolog (Trip13)	0.338	Required for recombination and meiotic chromosome structure (Roig et al., 2010)
Synaptonemal complex protein 3 (SYCP3)	0.256	Component of synaptonemal complex (Syrjänen et al., 2014)
Cohesin subunit SA-3 (STAG3)	0.173	Maintaining centromere chromatid cohesion; required for DSB repair and synapsis (Hopkins et al., 2014)
DNA mismatch repair protein Mlh1	0.146	Homologous recombination and DSB repair (Cannavo et al., 2020)
Synaptonemal complex protein 2 (SYCP2)	0.144	Component of synaptonemal complex (Feng et al., 2017)
Serine/threonine-protein kinase Chk1	0.137	DNA damage response (Nie et al., 2017)
Meiotic recombination protein REC8	0.124	Sister-chromatid cohesion and crossover recombination (Yoon et al., 2016)
HORMA domain-containing protein 1 (HORMAD1)	0.094	Synaptonemal complex formation, recombination and chromosome segregation (Shin et al., 2010)
Double-strand-break repair protein rad21-like protein 1 (Rad21L1)	0.038	Maintaining the integrity of meiotic chromatin architecture (Blokhina et al., 2020)
Synaptonemal complex protein 1 (SYCP1)	0.036	Component of synaptonemal complex (de Vries et al., 2005)
Serine-protein kinase ATM	0.026	Controlling DSB formation and recombination (Lange et al., 2011; Kurzbauer et al., 2021)

A growing number of *in vitro* experiments have been carried out to detect LLPS and study its underlying mechanisms and functions. First, *in vitro* phase-separation assay has been commonly used to study LLPS proteins. Second, engineered expression vectors containing fluorescence-tagged target proteins can also be utilized to study LLPS proteins in transfected cell lines, in which phase-separating behaviors could be visualized by fluorescent signals (Quiroz et al., 2020). Third, the self-assembly of phase-separating proteins *in vitro* can be visualized by conjugation and co-expression with fluorescent proteins, such as GFP. Fourth, approaches, including artificial modification of protein structures, changing of environmental conditions, and adding LLPS disruptors, have been used to study the dynamic features of LLPS proteins. For instance, 1,6-hexanediol, a disruptor of LLPS, is typically applied to determine whether LLPS plays a role in the formation of condensates both *in vivo* and *in vitro* (Ahn et al., 2020). Small molecules, such as kinases and ATPs, can also be added into *in vitro* phase-separation systems and exert specific biological functions (Mackenzie et al., 2017). In addition, fluorescence recovery after photobleaching (FRAP), fluorescence correlation spectroscopy (FCS), and fluorescence resonance energy transfer (FRET), are also frequently conducted to evaluate the properties of LLPS condensates.

Not only *in vitro* LLPS systems build our knowledge on the behavior of LLPS proteins, but *in vivo* studies also provided insights into LLPS droplets in cells. Endogenous phase behavior has been revealed by engineered phase-separation sensors. In the study of keratohyalin granules (KGs) in the mammalian skin barrier, modified variants of phase-separating intrinsically disordered proteins (IDPs) were fused to fluorescent proteins and acted as phase separation sensors after transducing them into mouse embryos (Quiroz et al., 2020). These IDPs do not exhibit phase-separation behavior on their own; in contrast, when exposed to phase-separated droplets, they are able to engage in phase-separation-specific interactions and report nascent phase-separating activities. As a novel method to investigate phase separation *in vivo*, the phase-separation sensor can potentially be applied to explore LLPS in other tissues or cells. Additionally, mutations that alter the phase-separating properties, such as protein multivalency, can also be introduced to examine the LLPS functions (Nott et al., 2015). For instance, the hnRNP protein, TIA1, contains a C-terminal LCD domain that is predicted to be intrinsically disordered. Three ALS-associated TIA1 mutations exhibit increased phase-separation tendency that is caused by stronger protein-protein interactions (Mackenzie et al., 2017).

Although LLPS is likely a mechanism to explain the sex-body formation, other models for assembling chromatin subcompartments should also be considered as reviewed by Erdel and Rippe (2018). First, the multivalent interactions between chromatin-associated proteins promote LLPS. In polymer-polymer phase separation (PPPS) model, bridges between nucleosomes, rather than multivalent interactions in LLPS, facilitate the formation of phase-separated subcompartments within polymers (Singh and Newman, 2020). The bridging factors crosslinked chromatin fibers are usually chromatin-associated proteins lacking multivalent interactions, such as condensin (Ganji et al., 2018), cohesin (Rao et al., 2017), and YY1 (Weintraub et al., 2017). Second, without phase-separation, the simple binding between soluble factors and the chromatin scaffold is also able to form the chromatin subcompartments (Wachsmuth et al., 2008).

Experimental strategies to distinguish different mechanisms were also proposed by Erdel and Rippe (2018). First, tracking the (dis)assembly process over time could distinguish LLPS and PPPS because PPPS formation but not LLPS need nucleation sites. Thus, introducing and subsequently removing artificial nucleation sites can help us test whether LLPS multivalent interactions can hold the sex body together without nucleation sites. Second, with a concentration increase of constituting protein factors in the nucleoplasm, the size of the sex body should increase via LLPS but not via PPPS. Third, bridging factors need chromatin scaffolds to form biomolecule condensates. However, multivalent binders in LLPS can independently form condensates without chromatin scaffolds.

Some membraneless organelles can also transit from liquids (young) to gel/solid-like state (old) or have solid-like substructures, such as centrosomes (Woodruff et al., 2017). After they mature, the hardened solid-like organelles reduce the dynamics of their molecules (Banani et al., 2017), e.g., some HP1 proteins become immobile during the maturation of the heterochromatin regions/domains (Strom et al., 2017). Similarly, sex bodies might also display different material properties from liquids to solid-like feature with “aging.” FRAP analysis can help us test this hypothesis (Boke et al., 2016) and show the “aged” sex body may not recover after full or partial bleaching. If it is true, the solid-like “aged” sex body loses its ability to incorporate and internally rearrange the fluorescence-tagged target proteins. This will be consistent with the idea that the hardened mature sex body serves as a sink to sequester its trapped proteins, including the DDR proteins, from the rest of the pachytene nucleoplasm (Abe et al., 2020).

## Concluding Remarks and Future Directions

Liquid-liquid phase separation is a driver for the assembly of membraneless biomolecular condensates in cells. It exerts a variety of functions, including sequestration of molecules, buffering molecule concentration, regulating the specificity and kinetics of biochemical reactions, genomic organization, RNA processing, and generating meiotic DNA breaks (Banani et al., 2017; Claeys Bouuaert et al., 2021). During male meiosis, sex chromosomes are reorganized, transcriptionally silenced by MSCI, and form a visibly distinct membraneless structure at pachytene stage. Although the molecular basis of MSCI has been extensively studied, it is not yet known what factors drive the formation of the sex body and mediate gene repression.

**FIGURE 5:**
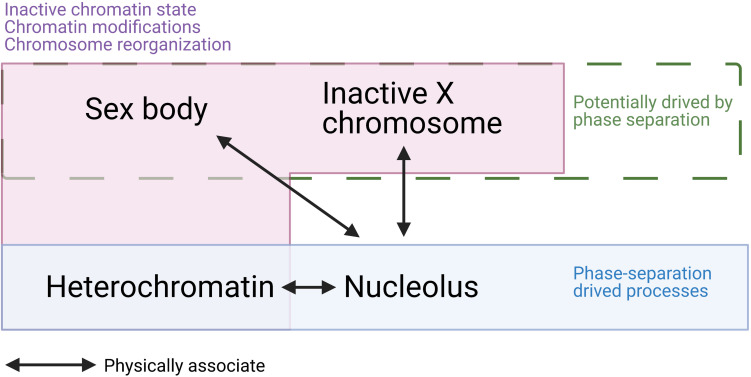
A summary of similarities and associations among sex body, inactive X chromosome (Barr body), heterochromatin, and nucleolus. Sex body, Barr body, and heterochromatin are all physically associated with the nucleolus. While heterochromatin and nucleolus are confirmed to be driven by phase separation, sex body and Barr body are hypothesized to be formed by LLPS. In addition, sex body, Barr body, and heterochromatin all present inactive chromatin state, dramatic chromatin modifications, chromosome reorganizations. These similarities and associations raise the possibility that sex body is formed by LLPS.

Here, we summarized several points that suggest there are similarities between the sex body and other phase-separation condensates and gene silencing processes (Figure 5). Firstly, the sex body has a similar appearance to other membraneless droplets formed by phase-separation and a separate territory that has a low interaction frequency with autosomes. Secondly, heterochromatin, which is structurally reorganized and transcriptionally silenced, is known to be formed by LLPS. Similarly, sex bodies also share the silencing status and histone modification with heterochromatin regions, such as H3K9me3. Thirdly, the phase-separating protein HP1 drives the formation of heterochromatin. Two isoforms of HP1, HP1β, and HP1γ, also localize to the sex body in spermatocytes (Metzler-Guillemain et al., 2003), indicating the resemblance of sex body and heterochromatin. Fourthly, MSCI also resembles XCI in female somatic cells. Both processes involve the recruitment of a set of proteins, histone modifications, chromosome reorganization, and transcriptional silencing. Cerase et al. (2019) gathered evidence and proposed a hypothesis that phase separation drives XCI. Overall, the similarities between sex-body formation and phase-separating processes raise the possibility that LLPS is a common driving force underlying all these processes—formation of the sex body, heterochromatin, and inactivation of the X chromosome.

Ever-growing experimental methods have been developed to study characteristics and mechanisms of LLPS, including both *in vivo* and *in vitro* approaches. Although growing clues indicate LLPS drives sex-body formation, so far, there is still no direct proof. In the future, approaches investigating LLPS in other cellular bodies could be applied to analyze sex-body formation. Softwares and tools analyzing protein sequences and phase-separating domains can be used to identify sex-body proteins involved in phase separation. Any identified phase-separating proteins can be further studied by *in vitro* or *in vivo* experiments. Established methods for visualizing the behavior of liquid condensates formed by LLPS will shed a light on the phase-separating process of sex bodies. Disrupting chemicals, such as 1,6-hexanediol, can be added to protein aggregates to determine whether phase separation will be disturbed. Phase separation sensors will enable the inspection of LLPS activity *in vivo.* Understanding the driving force and factors involved in sex-body formation is still challenging, however, new ways to explore LLPS characteristics will expand our knowledge on the properties of the sex bodies. By applying these methods to study proteins in the sex body, future studies are likely to identify many phase-separating proteins in the sex body. It is also intriguing to know how some X-linked genes can escape MSCI in these phase-separated sex bodies. We speculate those gene regions may locate at the phase boundary/the surface of the sex body and dissociate from phase-separating components. A challenge in the future is to understand how these “sex body” proteins drive the sex-body formation step-by-step and how the structure of the sex body is maintained until the diplotene stage in spermatocytes.

## Author Contributions

YX and HQ contributed to the writing and editing of this review. Both authors contributed to the article and approved the submitted version.

## Conflict of Interest

The authors declare that the research was conducted in the absence of any commercial or financial relationships that could be construed as a potential conflict of interest.

## Publisher’s Note

All claims expressed in this article are solely those of the authors and do not necessarily represent those of their affiliated organizations, or those of the publisher, the editors and the reviewers. Any product that may be evaluated in this article, or claim that may be made by its manufacturer, is not guaranteed or endorsed by the publisher.
